# Robot-Assisted Totally Intracorporeal Resection of Cutaneous Ureterostomy Tumor and Ileal Conduit Surgery: A Rare Case Report

**DOI:** 10.3389/fonc.2022.803221

**Published:** 2022-02-10

**Authors:** Lingkai Cai, Juntao Zhuang, Qiang Cao, Baorui Yuan, Qikai Wu, Kai Li, Jie Han, Hao Yu, JianCheng Lv, Dexiang Feng, Peikun Liu, Ruixi Yu, Pengchao Li, Xiao Yang, Qiang Lu

**Affiliations:** Department of Urology, The First Affiliated Hospital of Nanjing Medical University, Nanjing, China

**Keywords:** BCa, cutaneous ureterostomy, robot, recurrence, ileal conduit

## Abstract

**Background:**

Radical cystectomy (RC) is the standard treatment for muscular invasive bladder cancer (MIBC) and some high-risk non-muscular invasive bladder cancer (NMIBC). Cutaneous ureterostomy is a common form of urinary diversion. However, after radical cystectomy, recurrence of upper urinary tract malignancies is possible. There is no relevant report on how to improve this situation’s management.

**Case Presentation:**

This case is a 56-year-old male patient hospitalized due to the development of a new tumor in the ureteral cutaneous stoma following radical cystectomy for more than five years. A biopsy of the tumor revealed high-grade urothelial carcinoma. Computed tomography (CT) revealed that the local soft tissue around the cutaneous stoma was thickened, but no other lesions were visible. After evaluating the case, we chose robot-assisted completely intracorporeal resection of cutaneous ureterostomy tumor and ileal conduit surgery. The total time for the operation and the blood loss were 400 minutes and 150 ml, respectively. Following surgery, the patient got standard chemotherapy in combination with immunotherapy. Additionally, ten months following the surgery, the patient did not experience disease progression or complications.

**Conclusion:**

The robot-assisted operation is safe and feasible for upper urinary tract tumor recurrence following radical cystectomy with cutaneous ureterostomy.

## 1 Background

RC is the standard treatment for MIBC and some high-risk NMIBC (1, 2). The most commonly used procedures of urine diversion include cutaneous ureterostomy, ileal conduit and orthotopic ileal neobladder. Menon et al. described the robotic assistant radical cystectomy (RARC) for the first time in 2003 (3). Numerous studies have demonstrated that RARC is superior to laparoscopic radical cystectomy in terms of perioperative safety and oncological outcomes (4, 5). Although robot-assisted intracorporeal urine diversion is possible, a multi-institutional evaluation found that only 3% of RARC were performed with totally intracorporeal urinary diversion (6). Here, we present a case of a 56-year-old man who received robot-assisted totally intracorporeal resection of cutaneous ureterostomy tumor and ileal conduit surgery.

## 2 Case Report

### 2.1 Patient Information

A 51-year-old male patient was admitted to hospital with hematuria and underwent laparoscopic radical cystectomy and modified single cutaneous ureterostomy (CU) five years ago. The patient has no history of smoking or any comorbidities. CT scan indicated clinical staging of T2N0M0 and the preoperative cystoscopy revealed multiple masses in the bladder and posterior urethra. Subsequent pathology confirmed urothelial carcinoma. Due to the patient’s refusal of neoadjuvant chemotherapy, he underwent RC directly. The operation lasted five hours with 200 ml of blood loss. The pathology results indicated that the tumor invaded the bladder muscle layer and the prostate duct without metastatic lymph nodes. And the patient received three cycles of GC (gemcitabine and cisplatin) chemotherapy after surgery. And this patient’s postoperative course was free of serious complications.

**Figure 1:**
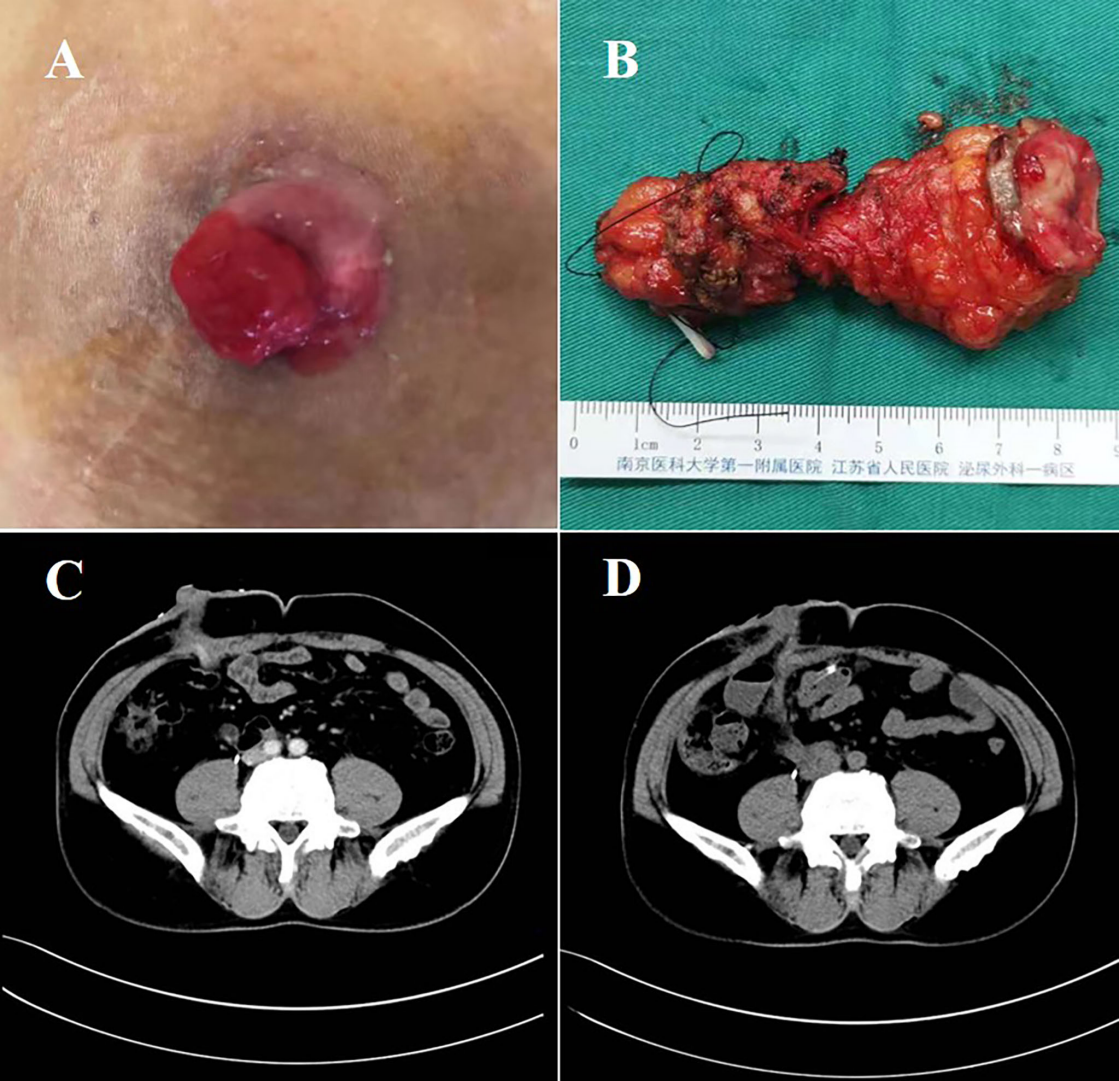
Surgical specimens and CT images. **(A)** The stoma mass of the patient before surgery. **(B)** The resected ureter after surgery. **(C)** CT image of the patients’ stoma mass before surgery. **(D)** CT image of the patients’ abdominal organs after surgery. CT, computed tomography.

After accidently discovering a mass in the ureteral cutaneous stoma, the patient was admitted to hospital after a biopsy confirmed the presence of a high-grade urothelial carcinoma (**Figure 1A**). The patient had no history of smoking, which was consistent with the RC preoperatively. As shown in **Figure 1C**, CT revealed that the local soft tissue around the cutaneous stoma was thickened, but no other lesions were visible. After evaluating the case, we chose robot-assisted completely intracorporeal resection of cutaneous ureterostomy tumor and ileal conduit surgery. The total time for the operation and the blood loss were 400 minutes and 150 ml, respectively. Postoperative pathology indicated that: “The tumor is an invasive high-grade urothelial carcinoma that infiltrates the entire ureteral wall.” Five days after surgery, the patient defecated, and creatinine levels in blood decreased from 183.2 umol/L to 119.3umol/L. Then single J tubes were removed one month later.

Following surgery, the patient received five cycles of nab-paclitaxel chemotherapy in combination with PD-1 immunotherapy, followed by PD-1 immunotherapy for maintenance. During the ten-month follow-up postoperatively, no evidence of tumor recurrence and complications was discovered (**Figure 1D**).

### 2.2 Techniques of Surgery

#### 2.2.1 Stage 1: Port Placement

**Figure 2:**
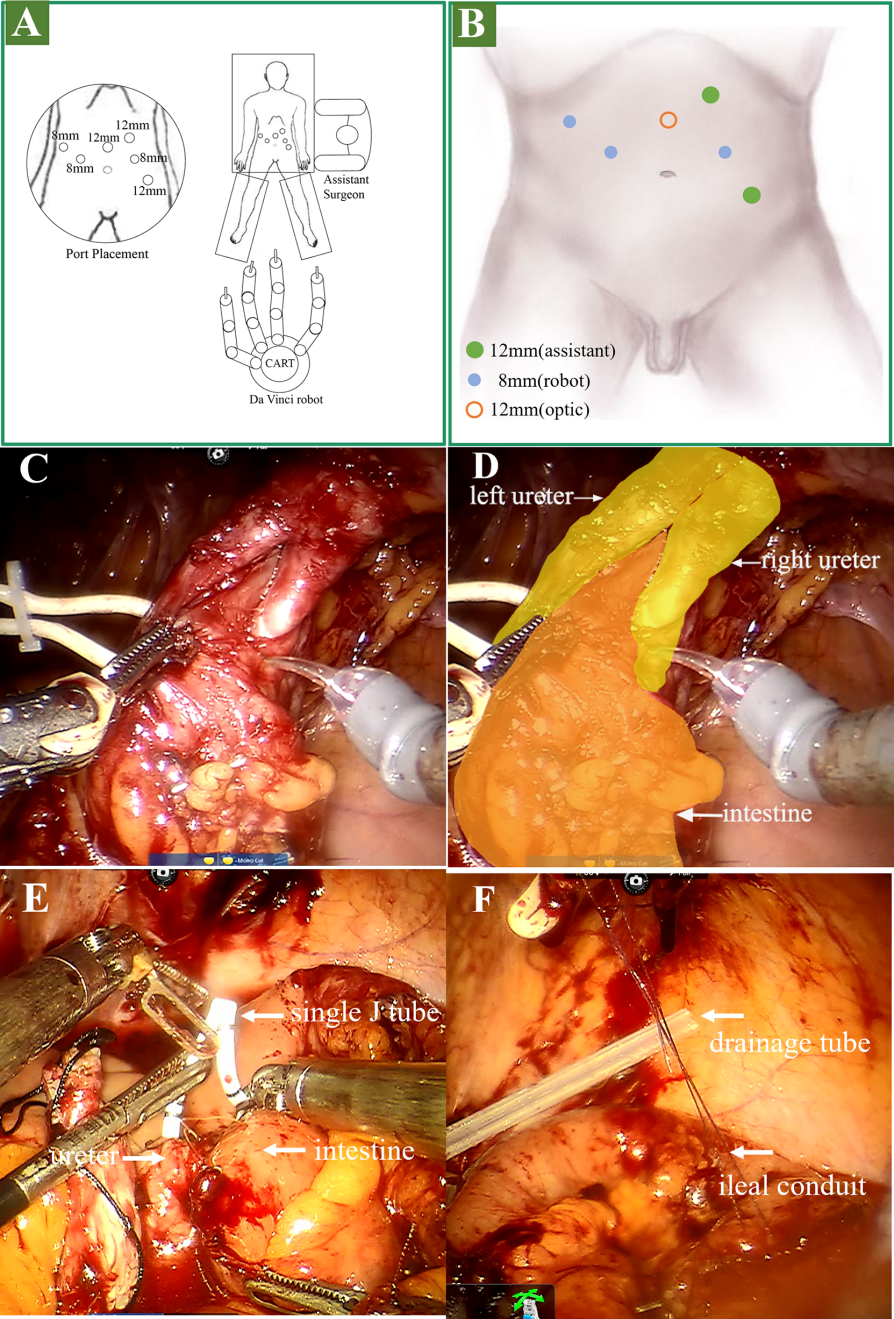
Operation procedure. **(A, B)** stage 1 surgical position and port placement. **(C, D)** stage 2: lysis of adhesions and dissociation of the ureter. **(E)** stage 3: placed the single J stent. **(F)** stage 3: suspended the outlet of the ileal conduit to the stoma.

All ports are raised above the usual 5cm. As shown in **Figures 2** **A, B**, a six-port technique was adopted. In brief, a 12-mm camera port was positioned above the umbilicus in the midline. The robotic ports were positioned 2cm above the umbilical, along the lateral margin of the rectus sheath. Both the right and left ports were positioned 8cm from the midline. The last 8-mm robotic instrument port was placed in the right anterior axillary line, 3cm below the right costal arch. Additionally, a 12-mm assistant port was positioned above and inside the left anterior superior iliac spine. And the other 12-mm assistant port was positioned 3cm below the left costal arch in the parasternal line.

#### 2.2.2 Stage 2: Lysis of Adhesions and Dissociation of the Ureter

As shown in **Figures 2** **C, D**, the location of the ureterostomy was determined. The adhesions around the stoma and in the pelvic cavity were separated, followed by the intestine surrounding the stoma was separated. Then the left and right proximal ureters were dissociated. To ensure that the margin was negative, we clipped the left and right ureter and incised the distal margin for frozen pathology.

#### 2.2.3 Stage 3: Establishment of the Ileal Conduit

We established the ileal conduit using the Bricker ureteroenteric technique. Cut the ileum 20cm from the ileocecal junction and extract the expected 12cm ileal conduit. The intestine was then reconstructed for continuity. The left ureter and ileum were then anastomosed. As shown in **Figure 2E**, the guidewire and single J stent were inserted into the left ureter, and the distal end of the single J stent was inserted into the ileal conduit. The right ureteroenteric anastomosis was performed in the same way. Then we suspended the outlet of the ileal conduit to the stoma (**Figure 2F**). Afterward, a drainage tube was placed in the coeliac beneath the stoma and removed six days following surgery (**Figure 2F**). Finally, we extracorporeally resected the stoma and 1cm skin around it, and cut the incised margin for frozen pathology (**Figure 1B**), followed by enterostomy of the ileal conduit. The video of the surgery is in the additional material.

## 3 Discussion

Bladder cancer is one of the common malignancies of the urinary system. Nathan et al. (7) analyzed 574 patients who received RC and discovered 3.7% probability of upper urinary tract (UUT) recurrence. The treatment method was reported in the patients with UUT recurrence, including nephroureterectomy, laparoscopic resection and segmental ureterectomy (8). We were the first to describe the detailed operation plan for a patient who developed ureteral cutaneous recurrence following RC.

The challenge with this type of surgery is the adhesion of the abdominal cavity following RC, which makes dissociating the ureter and intestine difficult. The robot’s assistance makes full use of its flexibility and safety characteristics during its operation.

Essentials of surgery: The patient received robot-assisted totally intracorporeal resection of cutaneous ureterostomy tumor and ileal conduit surgery. The robotic trocars were positioned 5cm closer to the head than the conventional position (9), allowing for more space for laparoscopic surgery. The intestines, ureters, and abdominal wall tissues were severely adherent as a result of the previous operation, and robot assistance is beneficial for loosening abdominal adhesions. After uretero-ileum anastomosis, the single J tube was placed under endoscopy. Sutured the ileal conduit outlet to the stoma, then substantially resected the tumor and prolapsed the distal end of the outflow tract to perform a new enterostomy.

The ileal conduit (IC) is currently considered the superior modality for the type of urinary diversion (10). However, despite the high risk of advanced ureteral strictures, CU has the advantages of a shorter operation time, less bleeding and fewer early complications for the elderly and weak patients (11). Additionally, there were no conclusive research demonstrating that CU patients have a lower health-related quality of life (HR-QoL) than those with ileal conduits (12). And the adoption of totally intracorporeal neobladder comes with many limitations, including patient status, tumor location, tumor stage, and so on (13). Therefore, numerous patients who were suffered from UUT recurrence, severe ureteral stricture or iterative UUT stones underwent RC with modified single CU or standard CU (14–16).

Thus, when UUT recurrence occurs following RC with CU, it is feasible and safe to adopt a robot-assisted tumor resection combined with IC on the patient who underwent RC with CU. Additionally, we can get knowledge about the therapeutic options for CU associated with ureteral stricture or iterative UUT stones.

## 4 Conclusion

The robot-assisted totally intracorporeal resection of CU tumor and IC construction was safe and feasible.

## 5 Patient Perspective

The patient felt that he had less injury by undergoing the robot-assisted operation. And there was no significant change in the position of the stoma after surgery from the previous one. Besides, there was no need for frequent replacement of the single J-tube, which made his life more convenient.

## Data Availability Statement

The original contributions presented in the study are included in the article/**Supplementary Material**. Further inquiries can be directed to the corresponding authors.

## Ethics Statement

Written informed consent was obtained from the individual(s) for the publication of any potentially identifiable images or data included in this article.

## Author Contributions

LC: made substantial contributions to the conception, design of the work, acquisition, analysis, and interpretation of data and has drafted the work. JZ and QC: made substantial contributions to the interpretation of data and have substantially revised it. QW and BY: made substantial contributions to the picture editing. KL and JH: made substantial contributions to the patient treatment and care. HY, JL, DF, PKL, RY, and PCL: made substantial contributions to the manuscript revision. XY and QL: made substantial contributions to the conception, design of the work, and analysis. All authors read and approved the final manuscript.

## Funding

This work was supported by the National Natural Science Foundation of China (grantsNo.82072832,81772711), the “333” project of Jiangsu Province (LGY2016002), and Jiangsu Province’s Key Provincial Talents Program (ZDRCA2016006).

## Conflict of Interest

The authors declare that the research was conducted in the absence of any commercial or financial relationships that could be construed as a potential conflict of interest.

## Publisher’s Note

All claims expressed in this article are solely those of the authors and do not necessarily represent those of their affiliated organizations, or those of the publisher, the editors and the reviewers. Any product that may be evaluated in this article, or claim that may be made by its manufacturer, is not guaranteed or endorsed by the publisher.
